# *N*-[2-(9*H*-Carbazol-9-yl)eth­yl]-4-(methyl­sulfon­yl)aniline

**DOI:** 10.1107/S1600536814003614

**Published:** 2014-02-22

**Authors:** Hongshan Lai, Judith C. Gallucci, Chenglong Li

**Affiliations:** a Division of Medicinal Chemistry and Pharmacognosy, College of Pharmacy, The Ohio State University, Columbus, OH 43210, USA; b Department of Chemistry and Biochemistry, 100 West 18th Avenue, The Ohio State University, Columbus, OH 43210, USA

## Abstract

In the title mol­ecule, C_21_H_20_N_2_O_2_S, the dihedral angle between the mean plane of the carbazole ring system [maximum deviation = 0.021 (4) Å] and the benzene ring is 80.15 (6)°. In the crystal, mol­ecules are linked by N—H⋯O and weak C—H⋯O hydrogen bonds into a *C*(8) chain along [001].

## Related literature   

For a related structure, see: Lai *et al.* (2014 ▶) For the synthesis, see: Abdel-Magid *et al.* (1996 ▶); Hallberg *et al.* (1982 ▶). For hydrogen bond graph-set notation, see: Bernstein *et al.* (1995 ▶).
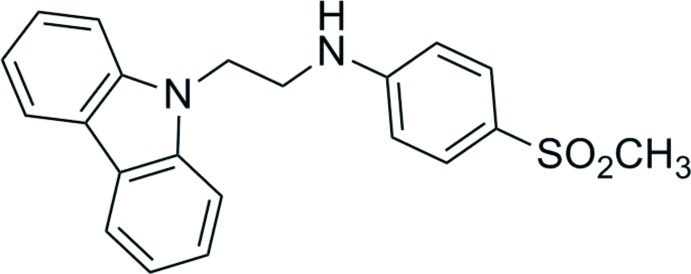


## Experimental   

### 

#### Crystal data  

C_21_H_20_N_2_O_2_S
*M* *_r_* = 364.45
Orthorhombic, 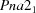

*a* = 15.061 (3) Å
*b* = 21.992 (4) Å
*c* = 5.437 (1) Å
*V* = 1800.9 (6) Å^3^
*Z* = 4
Synchrotron radiation
λ = 0.7749 Å
μ = 0.21 mm^−1^
*T* = 150 K
0.13 × 0.01 × 0.01 mm

#### Data collection  

Bruker APEXII diffractometer
Absorption correction: multi-scan (*SADABS*; Sheldrick, 1996 ▶) *T*_min_ = 0.635, *T*_max_ = 0.746
21673 measured reflections
4447 independent reflections
3950 reflections with *I* > 2σ(*I*)
*R*_int_ = 0.094

#### Refinement  

*R*[*F* ^2^ > 2σ(*F* ^2^)] = 0.045
*wR*(*F* ^2^) = 0.118
*S* = 1.05
4447 reflections
240 parameters
1 restraint
H atoms treated by a mixture of independent and constrained refinement
Δρ_max_ = 0.21 e Å^−3^
Δρ_min_ = −0.33 e Å^−3^
Absolute structure: Flack parameter determined using 1610 quotients (Parsons *et al.*, 2013 ▶)
Absolute structure parameter: 0.04 (6)

### 

Data collection: *APEX2* (Bruker, 2013 ▶); cell refinement: *SAINT* (Bruker, 2013 ▶); data reduction: *SAINT*; program(s) used to solve structure: *SHELXS2013* (Sheldrick, 2008 ▶); program(s) used to refine structure: *SHELXL2013* (Sheldrick, 2008 ▶); molecular graphics: *ORTEP-3 for Windows* (Farrugia, 2012 ▶) and *Mercury* (Macrae *et al.*, 2006 ▶); software used to prepare material for publication: *WinGX* (Farrugia, 2012 ▶).

## Supplementary Material

Crystal structure: contains datablock(s) global, I. DOI: 10.1107/S1600536814003614/lh5693sup1.cif

Structure factors: contains datablock(s) I. DOI: 10.1107/S1600536814003614/lh5693Isup2.hkl

Supporting information file. DOI: 10.1107/S1600536814003614/lh5693Isup3.cml

CCDC reference: 987458

Additional supporting information:  crystallographic information; 3D view; checkCIF report

## Figures and Tables

**Table 1:** Hydrogen-bond geometry (Å, °)

*D*—H⋯*A*	*D*—H	H⋯*A*	*D*⋯*A*	*D*—H⋯*A*
N2—H1*N*2⋯O2^i^	0.88 (4)	2.16 (4)	3.012 (3)	164 (3)
C21—H21*B*⋯O1^ii^	0.98	2.31	3.281 (4)	171

Symmetry codes: (i) 
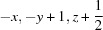
; (ii) 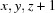.

**Fig. 1.**
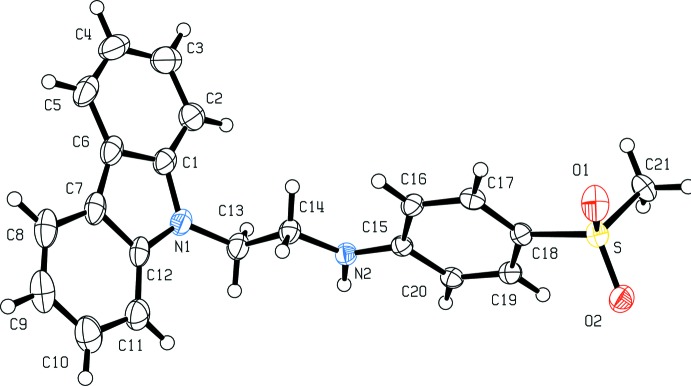
The molecular structure of (I) with displacement ellipsoids drawn at the 50% probability level (arbitrary spheres for the H atoms).

**Fig. 2.**
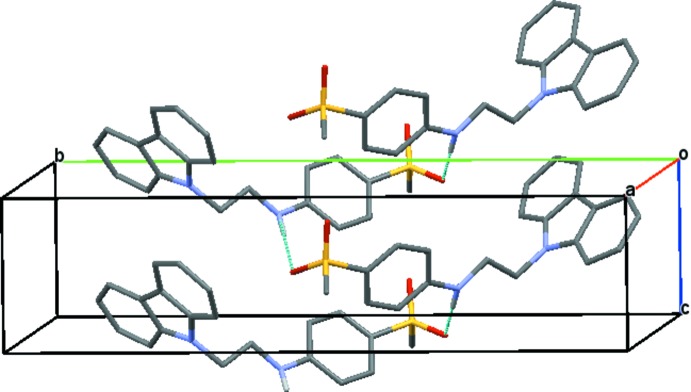
Crystal packing of (I) showing a portion of the unit cell emphasizing intermolecular N—H···O hydrogen bonds (dotted lines). Hydrogen atoms, except H(1N2), are omitted for clarity.

## supplementary crystallographic information

### 1. Comment

Carbazole based compounds are important in drug discovery. As part of our ongoing effort to develop bioactive carbazole compounds (Lai *et al.*, 2014), herein we report the crystal structure of the title compound (I). Compound (I) was obtained by reductive amination of carbazole-9-acetaldehyde through a direct procedure from Abdel-Magid *et al.* (1996). Carbazole-9-acetaldehyde was synthesized according to the method of Hallberg *et al.* (1982). The molecular structure of (I) is shown in Fig.1. A portion of the unit cell shown in Fig. 2 illustrates how the molecules are linked by strong intermolecular N—H···O hydrogen bonds. These hydrogen bonds form a one-dimensional chain along [0 0 1] with a graph set descriptor of C(8) (Bernstein *et al.*, 1995).

### 2. Experimental

All chemicals used were purchased from commercial sources and used without further purification. Carbazole-9-acetaldehyde (2.09 g, 10 mmol) and 4-(methylsulfonyl)aniline (1.71 g, 10 mmol) were mixed in 1,2-dichloroethane (35 ml), followed by the addition of sodium triacetoxyborohydrate (2.97 g, 14 mmol). The reaction mixture was stirred at room temporature under a N_2_ atmosphere for 12 h and then quenched by aqueous saturated NaHCO_3_. The resulted mixture was extracted with EtOAc, dried (MgSO_4_) and concentrated to give the crude product as nearly colorless oil, which was further purified by column chromatography (Hexane/EtOAc 1/1) to provide white solid (2.89 g, 79%). This solid was characterized by NMR to be the title compound. Crystals were grown from MeOH/H_2_O (50:1 *v*/*v*) solution by slow evaporation.

### 3. Refinement

For the methyl group, the hydrogen atoms were added in calculated positions using a riding-model with C—H = 0.98 Å and U(H) = 1.5*U*_eq_(C). The torsion angle, which defines the orientation of the methyl group about the C—S bond, was refined. The hydrogen atom bonded to N2 was refined isotropically. The rest of the hydrogen atoms were included in the model in calculated positions using a riding-model with C—H = 0.95 to 0.99 Å and U(H) = 1.2*U*_eq_(C).

### Crystal data

**Table:**

C_21_H_20_N_2_O_2_S	*F*(000) = 768
*M**_r_* = 364.45	*D*_x_ = 1.344 Mg m^−^^3^
Orthorhombic, *P**n**a*2_1_	Synchrotron radiation, λ = 0.7749 Å
Hall symbol: P 2c -2n	Cell parameters from 5294 reflections
*a* = 15.061 (3) Å	θ = 2.5–30.9°
*b* = 21.992 (4) Å	µ = 0.21 mm^−^^1^
*c* = 5.437 (1) Å	*T* = 150 K
*V* = 1800.9 (6) Å^3^	Needle, colorless
*Z* = 4	0.13 × 0.01 × 0.01 mm

### Data collection

**Table:**

Bruker APEXII diffractometer	4447 independent reflections
Radiation source: synchrotron	3950 reflections with *I* > 2σ(*I*)
Si-<111> channel cut crystal monochromator	*R*_int_ = 0.094
ω scans	θ_max_ = 31.1°, θ_min_ = 1.8°
Absorption correction: multi-scan (*SADABS*; Sheldrick, 1996)	*h* = −20→19
*T*_min_ = 0.635, *T*_max_ = 0.746	*k* = −29→29
21673 measured reflections	*l* = −7→7

### Refinement

**Table:**

Refinement on *F*^2^	Secondary atom site location: difference Fourier map
Least-squares matrix: full	Hydrogen site location: inferred from neighbouring sites
*R*[*F*^2^ > 2σ(*F*^2^)] = 0.045	H atoms treated by a mixture of independent and constrained refinement
*wR*(*F*^2^) = 0.118	*w* = 1/[σ^2^(*F*_o_^2^) + (0.0448*P*)^2^ + 0.0604*P*] where *P* = (*F*_o_^2^ + 2*F*_c_^2^)/3
*S* = 1.05	(Δ/σ)_max_ < 0.001
4447 reflections	Δρ_max_ = 0.21 e Å^−^^3^
240 parameters	Δρ_min_ = −0.33 e Å^−^^3^
1 restraint	Absolute structure: Flack parameter determined using 1610 quotients [(*I*^+^)-(*I*^-^)]/[(*I*^+^)+(*I*^-^)] (Parsons *et al.*, 2013)
0 constraints	Absolute structure parameter: 0.04 (6)
Primary atom site location: structure-invariant direct methods	

### Special details

**Table:**

Experimental. Intensity data were collected at 150 K on a D8 goniostat equipped with a Bruker APEXII CCD detector at Beamline 11.3.1 at the Advanced Light Source (Lawrence Berkeley National Laboratory) using synchrotron radiation tuned to a wavelength of 0.7749 Angstroms. For data collection, frames were measured for a duration of 1 second using omega scans with a frame width of 0.3 degrees out to a maximum 2theta value of about 60 degrees. The data frames were collected using the program *APEX2* and processed using the program *SAINT* within *APEX2*. The data were corrected for absorption and beam corrections based on the multi-scan technique as implemented in *SADABS*.
Geometry. All e.s.d.'s (except the e.s.d. in the dihedral angle between two l.s. planes) are estimated using the full covariance matrix. The cell e.s.d.'s are taken into account individually in the estimation of e.s.d.'s in distances, angles and torsion angles; correlations between e.s.d.'s in cell parameters are only used when they are defined by crystal symmetry. An approximate (isotropic) treatment of cell e.s.d.'s is used for estimating e.s.d.'s involving l.s. planes.
Refinement. For the methyl group, the hydrogen atoms were added at calculated positions using a riding model with U(H) = 1.5 * *U*_eq_(bonded carbon atom). The torsion angle, which defines the orientation of the methyl group about the C-*S* bond, was refined. The hydrogen atom bonded to N(2) was refined isotropically. It is involved in an intermolecular hydrogen bond with atom O(2). The rest of the hydrogen atoms were included in the model at calculated positions using a riding model with U(H) = 1.2 * *U*_eq_(bonded atom).

### Fractional atomic coordinates and isotropic or equivalent isotropic displacement parameters (Å^2^)

**Table:**

	*x*	*y*	*z*	*U*_iso_*/*U*_eq_	
C1	0.1214 (2)	0.82284 (13)	0.0581 (6)	0.0327 (6)	
C2	0.0477 (2)	0.84713 (15)	0.1764 (7)	0.0399 (7)	
H2	0.0219	0.8276	0.315	0.048*	
C3	0.0132 (2)	0.90105 (14)	0.0840 (10)	0.0506 (9)	
H3	−0.0374	0.9187	0.16	0.061*	
C4	0.0514 (3)	0.92970 (16)	−0.1175 (9)	0.0539 (11)	
H4	0.0268	0.9668	−0.1755	0.065*	
C5	0.1243 (3)	0.90545 (16)	−0.2349 (8)	0.0480 (10)	
H5	0.1496	0.9255	−0.3729	0.058*	
C6	0.1607 (2)	0.85076 (14)	−0.1479 (6)	0.0360 (7)	
C7	0.2347 (2)	0.81324 (15)	−0.2213 (6)	0.0354 (7)	
C8	0.2982 (2)	0.81655 (16)	−0.4086 (7)	0.0440 (8)	
H8	0.2967	0.849	−0.5239	0.053*	
C9	0.3627 (2)	0.77243 (17)	−0.4247 (8)	0.0502 (9)	
H9	0.4059	0.7746	−0.5516	0.06*	
C10	0.3653 (2)	0.72436 (18)	−0.2553 (8)	0.0494 (9)	
H10	0.4106	0.6945	−0.2692	0.059*	
C11	0.3031 (2)	0.71940 (15)	−0.0676 (7)	0.0404 (8)	
H11	0.305	0.6869	0.0473	0.048*	
C12	0.2379 (2)	0.76431 (13)	−0.0553 (6)	0.0317 (7)	
C13	0.1389 (2)	0.72389 (14)	0.2840 (6)	0.0328 (6)	
H13A	0.1136	0.7433	0.4327	0.039*	
H13B	0.1898	0.6984	0.3354	0.039*	
C14	0.06868 (19)	0.68418 (13)	0.1617 (5)	0.0269 (6)	
H14A	0.0188	0.7098	0.1041	0.032*	
H14B	0.0946	0.6633	0.0173	0.032*	
C15	−0.02888 (17)	0.59855 (12)	0.2799 (5)	0.0221 (5)	
C16	−0.08094 (17)	0.60306 (11)	0.0661 (5)	0.0255 (5)	
H16	−0.0722	0.636	−0.0442	0.031*	
C17	−0.14500 (18)	0.55956 (12)	0.0158 (5)	0.0246 (5)	
H17	−0.1804	0.5629	−0.1283	0.03*	
C18	−0.15764 (17)	0.51094 (13)	0.1759 (5)	0.0231 (5)	
C19	−0.10887 (18)	0.50682 (12)	0.3933 (5)	0.0246 (5)	
H19	−0.1192	0.4743	0.5049	0.03*	
C20	−0.04564 (18)	0.55020 (11)	0.4450 (5)	0.0235 (5)	
H20	−0.0128	0.5476	0.5938	0.028*	
C21	−0.32563 (18)	0.46030 (14)	0.2710 (6)	0.0309 (6)	
H21A	−0.3532	0.4995	0.2312	0.046*	
H21B	−0.3106	0.4593	0.4463	0.046*	
H21C	−0.3673	0.4273	0.2335	0.046*	
N1	0.16909 (16)	0.77046 (11)	0.1140 (5)	0.0316 (5)	
N2	0.03638 (16)	0.63949 (10)	0.3373 (5)	0.0266 (5)	
H1N2	0.072 (2)	0.6283 (16)	0.458 (8)	0.033 (9)*	
O1	−0.25201 (17)	0.45669 (11)	−0.1613 (4)	0.0384 (6)	
O2	−0.18686 (14)	0.39497 (10)	0.1746 (4)	0.0342 (5)	
S	−0.22847 (4)	0.45106 (3)	0.09511 (14)	0.02501 (17)	

### Atomic displacement parameters (Å^2^)

**Table:**

	*U*^11^	*U*^22^	*U*^33^	*U*^12^	*U*^13^	*U*^23^
C1	0.0351 (14)	0.0268 (13)	0.0363 (17)	−0.0085 (11)	−0.0104 (13)	0.0006 (12)
C2	0.0363 (16)	0.0383 (17)	0.0449 (18)	−0.0062 (13)	−0.0093 (14)	0.0000 (15)
C3	0.0440 (18)	0.0332 (15)	0.075 (3)	−0.0018 (13)	−0.020 (2)	−0.006 (2)
C4	0.051 (2)	0.0314 (16)	0.079 (3)	−0.0054 (16)	−0.033 (2)	0.0126 (19)
C5	0.051 (2)	0.0380 (17)	0.055 (2)	−0.0196 (16)	−0.0268 (18)	0.0199 (17)
C6	0.0396 (16)	0.0318 (14)	0.0365 (16)	−0.0144 (13)	−0.0123 (13)	0.0060 (13)
C7	0.0398 (17)	0.0363 (16)	0.0300 (15)	−0.0186 (13)	−0.0082 (13)	0.0045 (13)
C8	0.0500 (18)	0.0485 (18)	0.0336 (15)	−0.0260 (15)	−0.0034 (18)	0.0030 (18)
C9	0.052 (2)	0.055 (2)	0.044 (2)	−0.0253 (16)	0.0139 (19)	−0.010 (2)
C10	0.0450 (19)	0.046 (2)	0.057 (2)	−0.0137 (16)	0.0106 (18)	−0.0116 (18)
C11	0.0416 (17)	0.0318 (15)	0.048 (2)	−0.0100 (14)	0.0013 (16)	−0.0027 (14)
C12	0.0352 (16)	0.0296 (14)	0.0303 (15)	−0.0144 (12)	−0.0021 (12)	0.0013 (12)
C13	0.0346 (15)	0.0324 (14)	0.0314 (15)	−0.0075 (12)	−0.0042 (13)	0.0071 (12)
C14	0.0296 (13)	0.0272 (13)	0.0240 (13)	−0.0030 (11)	−0.0001 (10)	0.0033 (10)
C15	0.0219 (12)	0.0227 (12)	0.0217 (12)	0.0022 (10)	0.0025 (10)	−0.0006 (10)
C16	0.0274 (12)	0.0248 (12)	0.0244 (14)	0.0007 (9)	0.0009 (11)	0.0049 (11)
C17	0.0241 (12)	0.0293 (13)	0.0204 (12)	0.0039 (10)	−0.0008 (10)	−0.0008 (10)
C18	0.0221 (11)	0.0255 (12)	0.0217 (11)	0.0003 (10)	0.0032 (10)	−0.0012 (10)
C19	0.0260 (12)	0.0274 (13)	0.0206 (12)	0.0019 (11)	0.0036 (10)	0.0029 (10)
C20	0.0232 (12)	0.0276 (12)	0.0198 (12)	0.0021 (10)	−0.0014 (10)	0.0015 (10)
C21	0.0214 (12)	0.0408 (16)	0.0306 (14)	−0.0027 (12)	0.0032 (11)	−0.0024 (13)
N1	0.0343 (12)	0.0285 (11)	0.0320 (13)	−0.0062 (9)	−0.0019 (12)	0.0060 (11)
N2	0.0258 (11)	0.0275 (11)	0.0266 (12)	−0.0027 (9)	−0.0041 (10)	0.0058 (10)
O1	0.0463 (13)	0.0475 (14)	0.0214 (11)	−0.0148 (10)	−0.0013 (10)	−0.0018 (9)
O2	0.0349 (11)	0.0261 (10)	0.0414 (12)	−0.0012 (8)	0.0067 (9)	−0.0009 (8)
S	0.0255 (3)	0.0285 (3)	0.0210 (3)	−0.0031 (2)	0.0026 (3)	−0.0023 (3)

### Geometric parameters (Å, º)

**Table:**

C1—C2	1.389 (5)	C13—H13A	0.99
C1—N1	1.391 (4)	C13—H13B	0.99
C1—C6	1.408 (5)	C14—N2	1.454 (4)
C2—C3	1.388 (5)	C14—H14A	0.99
C2—H2	0.95	C14—H14B	0.99
C3—C4	1.388 (7)	C15—N2	1.369 (4)
C3—H3	0.95	C15—C16	1.406 (4)
C4—C5	1.377 (7)	C15—C20	1.414 (4)
C4—H4	0.95	C16—C17	1.386 (4)
C5—C6	1.404 (5)	C16—H16	0.95
C5—H5	0.95	C17—C18	1.392 (4)
C6—C7	1.443 (5)	C17—H17	0.95
C7—C8	1.399 (5)	C18—C19	1.395 (4)
C7—C12	1.405 (5)	C18—S	1.751 (3)
C8—C9	1.376 (5)	C19—C20	1.377 (4)
C8—H8	0.95	C19—H19	0.95
C9—C10	1.403 (6)	C20—H20	0.95
C9—H9	0.95	C21—S	1.760 (3)
C10—C11	1.389 (5)	C21—H21A	0.98
C10—H10	0.95	C21—H21B	0.98
C11—C12	1.394 (5)	C21—H21C	0.98
C11—H11	0.95	N2—H1N2	0.88 (4)
C12—N1	1.393 (4)	O1—S	1.444 (2)
C13—N1	1.453 (4)	O2—S	1.449 (2)
C13—C14	1.524 (4)		
			
C2—C1—N1	129.0 (3)	N2—C14—C13	109.4 (2)
C2—C1—C6	122.5 (3)	N2—C14—H14A	109.8
N1—C1—C6	108.5 (3)	C13—C14—H14A	109.8
C3—C2—C1	117.4 (4)	N2—C14—H14B	109.8
C3—C2—H2	121.3	C13—C14—H14B	109.8
C1—C2—H2	121.3	H14A—C14—H14B	108.2
C4—C3—C2	121.2 (4)	N2—C15—C16	122.8 (2)
C4—C3—H3	119.4	N2—C15—C20	118.6 (2)
C2—C3—H3	119.4	C16—C15—C20	118.6 (2)
C5—C4—C3	121.3 (3)	C17—C16—C15	120.2 (2)
C5—C4—H4	119.3	C17—C16—H16	119.9
C3—C4—H4	119.3	C15—C16—H16	119.9
C4—C5—C6	119.1 (4)	C16—C17—C18	120.1 (3)
C4—C5—H5	120.4	C16—C17—H17	119.9
C6—C5—H5	120.4	C18—C17—H17	119.9
C5—C6—C1	118.5 (4)	C17—C18—C19	120.5 (3)
C5—C6—C7	134.3 (4)	C17—C18—S	120.3 (2)
C1—C6—C7	107.2 (3)	C19—C18—S	119.0 (2)
C8—C7—C12	119.0 (3)	C20—C19—C18	119.5 (2)
C8—C7—C6	134.4 (3)	C20—C19—H19	120.3
C12—C7—C6	106.6 (3)	C18—C19—H19	120.3
C9—C8—C7	119.5 (3)	C19—C20—C15	121.0 (3)
C9—C8—H8	120.3	C19—C20—H20	119.5
C7—C8—H8	120.3	C15—C20—H20	119.5
C8—C9—C10	120.6 (4)	S—C21—H21A	109.5
C8—C9—H9	119.7	S—C21—H21B	109.5
C10—C9—H9	119.7	H21A—C21—H21B	109.5
C11—C10—C9	121.5 (4)	S—C21—H21C	109.5
C11—C10—H10	119.2	H21A—C21—H21C	109.5
C9—C10—H10	119.2	H21B—C21—H21C	109.5
C10—C11—C12	117.0 (3)	C1—N1—C12	108.7 (3)
C10—C11—H11	121.5	C1—N1—C13	124.1 (3)
C12—C11—H11	121.5	C12—N1—C13	125.8 (3)
N1—C12—C11	128.6 (3)	C15—N2—C14	122.4 (2)
N1—C12—C7	108.9 (3)	C15—N2—H1N2	115 (2)
C11—C12—C7	122.4 (3)	C14—N2—H1N2	118 (2)
N1—C13—C14	110.1 (3)	O1—S—O2	117.85 (15)
N1—C13—H13A	109.6	O1—S—C18	109.12 (14)
C14—C13—H13A	109.6	O2—S—C18	107.56 (13)
N1—C13—H13B	109.6	O1—S—C21	108.10 (16)
C14—C13—H13B	109.6	O2—S—C21	107.20 (14)
H13A—C13—H13B	108.2	C18—S—C21	106.46 (14)
			
N1—C1—C2—C3	179.1 (3)	C20—C15—C16—C17	−2.3 (4)
C6—C1—C2—C3	−0.1 (5)	C15—C16—C17—C18	−0.5 (4)
C1—C2—C3—C4	−0.5 (5)	C16—C17—C18—C19	2.8 (4)
C2—C3—C4—C5	0.7 (6)	C16—C17—C18—S	−172.1 (2)
C3—C4—C5—C6	−0.2 (5)	C17—C18—C19—C20	−2.2 (4)
C4—C5—C6—C1	−0.4 (5)	S—C18—C19—C20	172.7 (2)
C4—C5—C6—C7	−179.7 (3)	C18—C19—C20—C15	−0.6 (4)
C2—C1—C6—C5	0.5 (4)	N2—C15—C20—C19	−178.3 (2)
N1—C1—C6—C5	−178.8 (3)	C16—C15—C20—C19	2.8 (4)
C2—C1—C6—C7	−180.0 (3)	C2—C1—N1—C12	−179.9 (3)
N1—C1—C6—C7	0.7 (3)	C6—C1—N1—C12	−0.6 (3)
C5—C6—C7—C8	−0.7 (6)	C2—C1—N1—C13	12.9 (5)
C1—C6—C7—C8	179.9 (3)	C6—C1—N1—C13	−167.8 (3)
C5—C6—C7—C12	178.8 (3)	C11—C12—N1—C1	178.7 (3)
C1—C6—C7—C12	−0.6 (3)	C7—C12—N1—C1	0.2 (3)
C12—C7—C8—C9	−0.8 (5)	C11—C12—N1—C13	−14.3 (5)
C6—C7—C8—C9	178.7 (4)	C7—C12—N1—C13	167.2 (3)
C7—C8—C9—C10	0.0 (5)	C14—C13—N1—C1	77.2 (4)
C8—C9—C10—C11	0.3 (6)	C14—C13—N1—C12	−87.8 (3)
C9—C10—C11—C12	0.1 (5)	C16—C15—N2—C14	−11.9 (4)
C10—C11—C12—N1	−179.2 (3)	C20—C15—N2—C14	169.2 (2)
C10—C11—C12—C7	−0.9 (5)	C13—C14—N2—C15	178.3 (2)
C8—C7—C12—N1	179.9 (3)	C17—C18—S—O1	10.9 (3)
C6—C7—C12—N1	0.2 (3)	C19—C18—S—O1	−164.0 (2)
C8—C7—C12—C11	1.2 (5)	C17—C18—S—O2	139.8 (2)
C6—C7—C12—C11	−178.4 (3)	C19—C18—S—O2	−35.1 (2)
N1—C13—C14—N2	−177.6 (2)	C17—C18—S—C21	−105.5 (2)
N2—C15—C16—C17	178.8 (3)	C19—C18—S—C21	79.5 (2)

### Hydrogen-bond geometry (Å, º)

**Table:**

*D*—H···*A*	*D*—H	H···*A*	*D*···*A*	*D*—H···*A*
N2—H1*N*2···O2^i^	0.88 (4)	2.16 (4)	3.012 (3)	164 (3)
C21—H21*B*···O1^ii^	0.98	2.31	3.281 (4)	171

Symmetry codes: (i) −*x*, −*y*+1, *z*+1/2; (ii) *x*, *y*, *z*+1.
